# A Casual Review of the Work on the Appendix

**Published:** 1908-07

**Authors:** Willis Jones

**Affiliations:** Surgeon to Wesley Memorial Hospital, Atlanta, Ga.


### A CASUAL REVIEW OF THE WORK ON THE APPENDIX.*

BY WILLIS JONES, M. D., SURGEON TO WESLEY MEMORIAL HOSPITAL, ATLANTA, GA.

We find records of incisions for the purpose of draining abscesses in the right iliac fossa dating back as far as beginning of the Christian era, being about the year 50 B. C. The first recorded case of diseases of the appendix was reported by Mestivier in the year 1759, who incised and drained an abscess in the right iliac fossa in a man of 45 years of age. The patient died a few days later and an autopsy revealed a badly diseased appendix, and when opened, it was found to contain a rusty pin. From the time Mestivier reported his case up to the year 1824, six other cases of abscess in the right iliac fossa were reported,

* Read before Ocmulgee Medical Society, April 21, also Fulton County Medical Society, April 23, 1908.

where autopsy revealed advance disease, and the presence of foreign bodies in the appendix.

All of the reports, covering a period of 65 years, up to 1824 referred to the diseased condition found in the appendix, but considered it secondary to some lesion originating in the colon. In 1824 Louyer Villermay published an article in which he described inflammations of the appendix as definite disease. In 1827 Melier, another Frenchman, published a paper on the subject even more striking in detail than his colleague, Louyer Villermay, calling particular attention to the fact, that at autopsies, the pathology of the appendix had been much overlooked, and predicted the possibilities of surgery for its relief.

The accurate descriptions of Louyer Villermay and the well founded theories of Melier were not used by their contemporaries in the profession, because Dupuytren, the leading surgeon of his day, was too narrow to realize the importance of this great opportunity, and remained an exponent of the thought of his time, which referred to the colon, the origin of all right iliac inflammatory processes. The German surgeons in applying the name Perityphlitis to all inflammations of the right iliac fossa, both encouraged and confirmed the erroneous opinion which has in some quarters hardly been obliterated in our own day.

The interpretation of the symptoms and pathological findings of inflammations in the right iliac fossa had kept pace with the rapid evolution of surgery, and were now recognized and known to be characteristic of disease of the vermiform appendix.

In 1884 Kronlein, of Germany, made a diagnosis of perforation of the appendix, and did the first operation for its removal. The wound was closed up without drainage and the patient died three days later. Two years afterwards, in 1886, Hall, of New York, did the first successful operation for the removal of the appendix with the recovery of his patient. Until this time dispute and contention was still rife as to the origin of abscesses in the right fossa, and not until Reginald Fitz, of Boston, in the same year gave to the surgical world his epoch making paper, clearly defining relationship between disease of the colon and appendix, did the subject which had been buried under a mass of inco-ordinate facts and unstable theories, become cleared of all obscurity, and established upon a scientific basis.

In this notable paper of his, we note the following dictum.

"It is the duty of every physician to be mindful that for all practical purposes, Perityphlitis, Perityphlitic Tumors, Perityphlitic Abscesses, mean inflammation of the vermiform appendix." And it is to Fitz we give the distinction of having named the condition Appendicitis, he preferring the term rather than having it known as Fitz' disease. After the atmosphere had been cleared by Fitz' paper, it was not long before our aggressive surgeons realized the misdirection of their former efforts, and were hasty in their attempts to handle this important condition, along the lines suggested by modern surgical pathology.

In 1889 McBurney gave a powerful stimulus to the surgical treatment of Appendicitis by demonstrating the feasibility of doing the operation without destroying the continuity of the abdominal wall. Since that time the McBurney inter-muscular operation has been the standard—others simulate, but none excel.

From this casual review of the literature on the subject of Appendicitis, we readily see that the aggressive surgery of the appendix as it is practiced today, is only 24 years old, and though first suggested by a Frenchman, first executed by a German, all praise be to our American surgeons, who have brought the work up to its present high standard of perfection.

The anatomy of the appendix is very interesting, and at times most perplexing, for its anatomical position is not fixed; in fact there is no portion of the abdominal cavity where the appendix may not be found. These anomalies of position are due first, to an abnormally long Meso-appendix; second, to an arrest of foetal development. A long appendix with a long Meso-appendix may extend across the media line of the body into the left iliac fossa, or, extend upwards or inwards among the coils of the intestine, and it has been found firmly attached to the anterior abdominal wall. When the cecum and appendix are found out by the right lower quadrant, this condition is due to an incomplete rotation of the gut during the process of foetal development, likewise, the cases of retrocaecal and retropetritoneal appendicitis, are due to faulty rotation. The average length of the appendix is 3 1-2 inches, but it has been found as long as 9 inches and as short as 3-4 of an inch. Its lumen or cavity is as variable as its length. It is found to be partially or totally occluded in at least one-fourth of all specimens examined. This variability of the organ as to position, size and structure, are considered by the evolutionist as a sign of degeneracy in the process, which is undergoing a gradual obliteration in the higher animals. Alimentation has an important bearing on the development of the cecum in many of the higher animals. Herbivorous animals have an enormously developed cecum, whereas, in carnivorous animals it is found small, and sometimes wanting. In man, apes, and many of the rodents where the food is midway between that of the Herbivorous and Carnivorous types, a retrogade development of the cecum is found, giving rise to a long worm-like projection from the base of the cecum which is known as the vermiform appendix. This retrogression is probably due to changes in the character of food which has taken place during the history of the species. Our foremost stu dents in comparative anatomy believe that the appendix is destined for further reduction in size and ultimate elimination.

What is the etiology of inflammation of this little troublesome worm-like organ? Naturally, we look to the various micro-organisms as being the exciting factors, chief among which is the colon baccillus. In connection with the exciting causes the predisposing must not be lost sight of. It must be borne in mind that the appendix is a vestigial organ undergoing an evolutionary and retrograde metamorphosis, and it is recognized that organs of this structure are especially liable and susceptible to inflammation. The scarcity of its blood supply renders it a poor combatter of infection. The appendicular artery, a branch of the ilio-colic, lying between the folds of the meso-appendix is very prone to circulatory disturbances from many conditions either physiological or pathological that increase intro-abdominal pressure. Acquired or congenital narrowing of its lumen precludes complete emptying of the organ, and fecal stagnation easily results. It is surrounded by a firm unyielding peritoneal coat, embarcing structures composed of soft embryonal elements, so that in an infection we have added compression and tension, which explain the rapidity with which gangrene may take place. With all these added predisposing anatomical factors, we should not wonder that appendicitis is the most frequent of all abdominal surgical diseases. The records of reporte dcases show that the disease is more frequent between the ages of ten and thirty years, during that period of life when lymphoid tissue is most abundant. Erdmann in his recent writings on Appendicitis, in children, published in the New York Medical Record reports, 100 cases varying in age from 21 months to 15 years, and in this series he was able to demonstrate foreign bodies in 41 of the 100 appendices removed. It is thought to be more frequent among men than women, because of the better blood supply in the female, the appendix sometimes receiving a branch from the ovarian artery. Digestive disturbances, constitutional disorders, movable kidney, foreign bodies, and trauma, have all been written about and discussed as possible predisposing agents. However numerous the predisposing cause, we should ever bear in mind that the essential for the production of an inflammation is the presence of someone or more of the pyogenic micro-organisms.

Pathologically, we find all varieties of lesion from the mild catarrhal to the fulminating and gangrenous types.

The early symptoms of the disease are not indicative of what the ultimate outcome may be. We have nothing to tell us which case is going to do badly and which one is going to do well. Pain and tenderness are the symptoms that are most often met with. Temperature elevation, and acceleration of the pulse are not indicators of the severity. Vomiting by some is thought to be a forerunner of Peritonitis. Rigidity of the right rectus muscles is usually present and most significant. Constipation is the rule, though diarrhea may exist. With all the symptoms of an acute inflammation in the appendix, without one single symptom to tell us whether the condition will go on to resolution or become gangrenous or perforate, what are we to advise our patients to do when consulted? Worcester, of Waltham, Mass., in a paper published in 1892, answers this question most empatically in the following words: "Appendicitis is an inflammation of a useless organ, dangerously situated. At the beginning of an attack it is not possible to determine whether it will prove of the harmless or of the dangerous type. The diagnosis is easy in comparison with the task of diagnosticating the seat of any acute inflammation. At the beginning of an attack the excision of the appendix is an easy and perfectly safe operation. If so treated, all complications and all subsequent attacks are avoided. In view of the results already obtained by following this treatment, no other treatment is worthy of consideration." From the time of his writings to the present day no one has been able to give more truthful and life saving advice covering this condition. Ask the surgéons the cause of mortality in their operations for appendicitis, and they will invariably tell you complications of delay. There is only one logical treatment for the disease that cannot be criticised, and that is, early excision of the organ as soon as the diagnosis is made, provided there are no heart, kidney or pulmonary contra-indications. What we wish to accomplish in the treatment of this condition is not to save one-half of our patients, nor four out of five, but all of them—and in order to do this, no chances of hopeful expectation must be taken, nor delays advised.

We must admit that 80 per cent. of all cases will recover under the so-called medical treatment, but the remaining twenty per cent. will do badly, and it is this twenty per cent. that we are striving to save. Of the 80 per cent. that respond to medical measures, a large number will at some future date have a return of the trouble, and this time may not yield to former treatment. Until the art of prognosis is established upon a more scientific plane, we had better be over-cautious in giving an opinion as to the ultimate outcome of any acute attack of appendicitis, for, in cases where we least expect it, we oftentimes find a pocket of pus or an appendix already gangrenous, and the patient having given us physical signs of neither. Research in the examination of the blood in these conditions has given to us some light upon the resistance of our patients, the fight they are making against the infection, and the degree of severity of the pathological process. I know of no disease where such discrepancies exist between physical signs and pathological findings as we often see in appendicitis.

In the simple catarrhal types of the disease, many surgeons give reports of mortality of less than half of one per cent. where several hundred cases have been operated upon successively. So gratifying are the results in the interval cases or in the early beginning of a disease that there was quite a tendency a few years ago to wait and watch for the interval, but since we are growing older in the work, we are likewise becoming wiser. Waiting and watching should not be taught or practiced either in private or public, for perforations occur under our own eyes, and the best clinicians in their endeavor to check spreading infection are confronted with disastrous results. Most surgeons will tell you that they are always worried while watching and waiting on a case of appendicitis not operated upon. Perforation and spreading infection kills ten times as many patients as inexperienced operators, in the early stages of the disease.

It has been urged that in grave cases abstention will save more lives than operation. Simpson reports thirteen cases of general or extensive Peritonitis, with only one death. Can abstension promise more? Surely nothing can be lost by providing an escape for an inflammatory exudate, thereby reducing the task of the body in taking care of the baccilli and the toxins which they produce. The main principal of the operative treatment in grave cases should be to do the least that would be sufficient to accomplish the object, whether it be to relieve the appendicitis or arrest a beginning of Peritonitis. The work should be done as rapidly as possible, ever mindful of doing the "minimum surgical trauma to accomplish the maximum of surgical safety." This having been done, I contend, will save more lives than similar cases treated along so-called conservative medical lines. As yet I know of no drug that will in any way stay the progress of beginning gangrene, or in the least lessen the accumulation and dissemination of its pathological products.

By the operation we strive to remove the appendix and establish drainage, if necessary. Choice of operation is usually a personal equation of the operator, some preferring McBurney's incision, some Deavers, and others the incision of Kammerer. Not so much is dependent upon the choice of incision as is upon the manipulation of the viscera adjacent to the diseased appendix. Here we should use all care and gentleness, ever careful not to break up adhesion, if pus or other inflammatory exudate be present. The appendix should be isolated, its mesentery securely tied, and its union with the base of cecum brought full in view. Various methods, all good when properly executed, for treating the stump are in vogue. Some ligate the appendix, cut and cauterize the stump and drop back into the abdomen, others after tying off the appendix, cutting it away invaginate the stump in the wall of the cecum and suture the peritoneum over its site; one method proving as satisfactory as the other in the hands of various skilled operators.

If called upon to operate after perforation has taken place, and we are reasonably sure of the presence of pus, Deaver's or Kammerer's incisions will probably find more favor than McBurney's, for they give more room for thorough investigation.

In such cases if we are convinced that the process is thoroughly walled off by adhesions, and the appendix difficult to locate, and if by endeavoring to isolate and separate it from the mesentery or coil of intestine to which it is adherent, we are in immediate danger of breaking down nature's barrier and spreading the contaminating exudate, it is far more advisable to establish thorough drainage in this class of cases and wait for the subsidance of the inflammation, before any attempt is made to remove the offending part. This necessarily means a second operation, but will leave a monument to our effort in the existence of our patient.

Every case where there is on exudate resulting from inflammation in the appendix should have some form of drain. In such a practice, many cases will be drained, that would recover without; but the uncertainty of some cases in which its absence might mean added danger, a second operation, or even death leads me to use it in every case where any doubt exists. Its disadvantages are not more than minor inconveniences. A slight delay in union, a transient pain on removal. In this operation, as in all others done in the abdominal cavity, the wound should be closed in layers, careful of leaving no dead space for the formation of clots, which so frequently are responsible for the failure of primary union.

Wonderful and marvelously progressive has been the science of medicine during the past quarter of a century. In the day of our own recollections we have either seen or heard of patients dying in a very few hours from an acute cramp colic, inflammation of the bowels, an acute typhoid fever, an acute congestive chill, and idiopathic peritonitis. Postmortem findings and pathology on these subjects, teach us that such conditions do not exist per se, but that in the appendix we find the destructive lesion. Rapidly we are having such misconceptions and erroneous diagnosis forever erased from the instructive pages of our modern medical literature. The operative mortality in appendicitis is going to be reduced, just with the same degree of rapidity as our patients learn to have the work done during the hours of election and not waiting until the time that necessity demands.

---

References:

Wyeth. Transactions of section on Surgery and Anatomy

A. M. A., 1907.

Price. Amer. Jour, Obs't's., Vol. LIV.

Scott. Amer. Jour. Obs't's., Vol. LIV.

Kelly and Hurdon. The Vermiform Appendix and its diseases.

Hurtington. Anatomy of Peretoneam and Abdomen.

Bissell. Hed. Record, Vol. LXXII.

Erdmann. Med. Record, Vol. 71.

Douglas. Surgical Diseases of Abdomen.

Stimson. Annals of Surgery, Vol. XLVI.

McBurney. N. Y. Med. Jour., Vol. I.

DISCUSSION.

*Dr. F. W. McRae.*—Enthusiastic as I am for surgery, ever ready to wield the scalpel that promises quick and sure relief, I cannot unqualifiedly endorse Dr. Jones' position, i. e., "That appendicitis, in the absence of complications, is always a surgical disease properly treated only by immediate operation, whether the diagnosis be made early or late." To me such a position seems illogical in the face of the inherent tendency of the disease towards recovering in eighty to ninety per cent. of first attacks. A disease of such protean types seen under such varying conditions, must admit of the intelligent application of medical and surgical principles.

What a relief it would be to me to know that my duty to every victim of appendicitis had been fully performed when I had recommended immediate operation by a competent surgeon! Alas, for my peace of mind, I cannot do so! I feel compelled to deal with each case of appendicitis on its merits. I have lain awake o'nights both on account of advising and on account of not advising immediate operation. I am a most earnest advocate of early diagnosis and immediate operation in all cases seen within the first 24 hours, practically all seen within the first 48 hours of the progress of the disease.

Many mild attacks of appendicitis will have practically subsided within 48 hours. I should hesitate about advising immediate operation in all such cases. I see some cases of appendicitis between the third and tenth days of the disease where the pathology is definitely localized, and well walled off as evidenced by the physical signs and general symptoms, where I advise delay and the Ochsner, starvation, plan of treatment. Such cases should be seen often and observed closely by a competent surgeon. It is far safer to drain an adherent, virtually extra-peritoneal, abscess on the eighth or tenth day than to expose the peritoneum to the virulent infection lurking in or about a very intensely inflamed appendix on the fourth or fifth day of the progress of the disease. I have had abscess cases brought to me from a distance, and found on arrival that the large tumor present before leaving home, had disappeared, the abscess having emptied through the bowels. I take it, no prudent surgeon would advise immediate operation under such conditions.

These are some of the exceptional conditions under which I do not advise immediate operation. I earnestly advocate immediate operation in all cases of spreading or general peritonitis except those practically moribund, removing the offending appendix with the greatest possible dispatch and the least possible handling and trauma, large properly placed drains through counter punctures, if need be, the Fowler position in bed and the Murphy normal salt solution procto-clysis.

My views on the treatment of appendicitis are based upon an experience comprising 528 appendectomies, with a total mortality including all deaths due to complications of every kind, of eighteen. I have done 398 interval operations of various types including recurring, chronic relapsing, and cold abscesses, with one death a mortality of partically 1-4 of 1 per cent. I have had two deaths in thirty-four acute cases, early operation, one from a co-existent double septic nephritis operated on at the same time, draining both kidneys. The other from apoplexy in a fat, elderly woman, on the fifth day, apparently from getting out of bed while the nurse was not in attendance. The remaining fifteen deaths were in acute abscess cases, and fulminant or gangrenous appendicitis with spreading or general perotinitis, fifty-three of the former and forty-two of the latter type.

In conclusion, gentlemen, I wish to state that I consider appendicitis first, last and all the time a surgical disease, but in such exceptional instances as those noted above not immediately an operative one.

*Dr. Cyrus Strickler.*—I believe in operating upon all cases of appendicitis. I don't understand why one should hesitate. I have never yet seen a case die from an operation, that would not certainly have died without it. The earlier the operation, the better chance we have of saving these cases. I believe an operation, at any time, safer than medical treatment.

*F. G. Hodgson.*—I advocate operating upon all cases just as soon as one is absolutely *sure* the patient has appendicitis. Delays are too dangerous, for we can not tell by the symptoms just what is going on in the appendix. If an abscess forms it should be drained at once, for while an occasional abscess ruptures into the bowel, they are more apt to rupture into the peritoneal cavity and cause a general peritonitis. I think many deaths from operation for appendicitis are due to too much manipulation and breaking up of adhesions.

I know of a case entering St. Luke's Hospital, N. Y., which was practically moribund, he was too weak to take an anaesthetic, so an incision was made under cocain over the appendix and the pus drained out. Another opening was made in the median line and drained. The patient began to improve the next day and made an uneventful recovery. So I think all cases should be given a chance. A simple incision with draining will cause very little shock and will assist nature in getting rid of the toxic materials. If the appendix is readily found, of course it should be removed.

*Dr. E. C. Davis* endorsed Dr. Jones' paper and emphasized the need of operation in all cases not contraindicated by pulmonary, renal or cardiac troubles. He said he was unable to tell what is going on in the abdomen by the external signs. No one knows the point to which the disease has progressed until in bad cases, it is often too late. Therefore there should be careful, judicious operating at once and the conditions met as they present themselves. He feels safer in this way than he does in trying to guess what is occuring in an abdomen.

*Dr. Sellman* asked as an anaesthetist, to be informed of the exact contraindicating conditions. His experience has been that the surgeon has gone ahead anyway, stating that the relief of the abdominal condition was more important than the risk of the contraindicating trouble.

*Dr. James N. Ellis.*—The recognition of surgical intervention as the proper procedure in the treatment of appendicitis, coincides, in date, with the beginning of my professional life. Consequently, with the advent of my first case of appendicitis, a consideration of the then unsettled question of when to operate, was immediately forced upon my attention. I still remember my anxiety in the treatment of this first case, and of those immediately succeeding it; of my indecision as to whether the time for surgical intervention had arrived, and of my subsequent unrest when I left the patient, fearing I was procrastinating, and had allowed the psycological surgical moment to pass.

Happily for my present serenity of mind and, I firmly believe, for the welfare of my patients, this period of perturbation, of uncertainty and of indecision on my part is long since a thing of the past. As a result of a continuously increasing experience and observation, my views on this vexed question have become thoroughly crystalized into a conviction that practically every case of appendicitis demands immediate operation as soon as the nature of the trouble is recognized, and competent surgical intervention can be obtained. The possible exception to this rule would be in the case of practically moribund patients, upon whom the operation, to use an Irishism, amounts to an ante-mortem post-mortem.

Appendicitis is a treacherous disease, and should be dealt with by the surgeon vigorously, aggressively, decisively and promptly. Delay in supposedly convalescing patients is fraught with danger and, often, with disaster. All of us who have had extended experience with appendicitis, and who, in our days of doubt have practiced expectant treatment, have seen such apparently convalescing patients suddenly and unexpectedly develope a general peritonitis, say from the eighth to the twelfth day, and die promptly, with or without surgical intervention. I have frequently operated on an apparently convalescing patient, and found an appendix ballooned to its utmost capacity with pus, and only the thin, serous investment intervening to protect the peritoneal cavity from a virulent, lethal infection.

The question with me now is not whether or when to operate, but what intervention shall I practice in this particular case? This frequently cannot be positively determined until the abdomen has been opened, and the exact pathological condition disclosed. So far as the surgeon is concerned, all cases of appendicitis may be resolved, for practical purposes, into four groups; namely, First, Chronic Appendicitis; Second, Acute Appendicitis, without involvement of adjacent structures; Third, Acute Appendicitis, with circumscribed abscess; and Fourth, Appendicitis, with diffuse peritonitis.

The technique of operation in groups one and two are practically the same, the McBurney muscle splitting, short incision being generally used to gain access to the cavity.

There is one method of dealing with the stump of the appendix in these cases, which I wish to mention in order to condemn it. I refer to the plan of burying the ligated, infested stump of the appendix by a superimposed purse string Suture of linen or Pagenstecker. The pocket thus made will necessarily become infected in a number of cases. While this does not kill, as the resulting abscess finally discharges into the caecum when the cat-gut ligature gives way, it is responsible for a temperature of 100 degrees or 101 degrees, not infrequently observed as late as the seventh or eighth day after operation by this method.

Operation in groups one and two is admittedly one of the safest and most satisfactory in surgery, and should be almost devoid of mortality.

In the third group, where the infection has extended to the neighboring structures, with the formation of a circumscribed abscess, immediate operation is, in my opinion, just as imperative, whether the attack has lasted twelve hours or twelve days, but the procedure is varied according to the individual findings.

The abdominal incision is made over the most prominent part of the tumour, and if the abscess wall is found adhering to the underlying peritoneum, the pus is evacuated without entering the general cavity; the appendix removed, if readily accessible, but left, if difficult of access, and drainage established. If the abscess is not adherent to the anterior abdominal wall, the surrounding mass is carefully isolated by gauze pads, effectually protecting the general cavity; the pus carefully sought for and gently removed by sponging; the appendix found and removed; drainage instituted; the abdomen closed and the patient put to bed in the exagerated Fowler position.

It is especially in the fourth group, namely those with diffuse peritonitis, in which delay in operating and the institution of the Ochsner method of treatment is being quite extensively and enthusiastically advocated by both surgeons and physicians. But surely, if the surgeon confines himself to making an outlet, or outlets from the peritoneal cavity for the escape of the accumulated purulent material, and provides for the temporary continuance of drainage, he has greatly facilitated the recovery of such a patient. It is after the establishment of such drainage that the inauguration of the Ochsner method of gastric lavage, prohibition of food by the mouth and rectal alimentation become life-saving measures, and find their logical applicability.

The Ochsner method, combined with the continuous saline enema of Murphy, and the semi-erect posture of Fowler, applied as a post-operative prodecure, is of incalculable value. As a substitute for immediate operation in appendicitis, it is deceitful and misleading, and, I believe, has proven a curse, by offering a seemingly safe course for timid patients, and procrastinating, vaccillating, temporizing, undecided surgeons.

*Dr. R. H. Donaldson.*—Practically every man doing abdominal surgery has his own ideas about when to operate in appendicitis, and my own personal opinion has changed several times, especially during my first few years of practice. I began with the idea of advising operation in all cases whenever seen. After some little while I came to the conclusion that each case should be treated on its individual merits and judgment used with regard to recommending operation. For the last four years I have recommended operation in all cases, whether seen early or late, and although I am sure that a great many cases would have recovered from the attack without operation, I believe that it is safer, more scientific, and will have a very satisfactory effect on one's mortality rate. It eliminates the excessive worry over each case, absolutely safeguards waiting too long, which is very important, because this organ is very like the human heart as described by David in being "deceitful above all things and desperately wicked."

I would possibly still be on the fence in regard to some exceptional cases, were it not for the fact that our technique has so improved that with rapid, successful work, very little handling, a conservative amount of drainage and the most excellent after-treatment (to which several gentlemen have referred tonight), we can usually handle the situation, it matters little what pathological condition may be present.

I most heartily endorse everything in Dr. Jones' excellent paper.

*Dr. W. S. Goldsmith.*—The discussion of this paper has assumed a wide latitude, going from the treatment of simple catarrhal appendicitis to perforative peritonitis. I am in entire accord with the views expressed relative to the treatment of the extreme types of the disease; that is, I favor early operation and the treatment of peritonitis cases by the Fowler position and the Murphy instillation. The importance and value of this latter treatment admits of no argument. I shall now repeat the assertion, made upon this floor two or three years ago relative to cases of three to five days duration, which are in an active abscess formation period, where the temperature is running from 101 to 103 and pulse 110 to 115, that this is the stage where under no circumstances do I operate. I am really surprised at the views taken by the gentlemen discussing this subject tonight, that they would, without reservation, operate on all cases of appendicitis pust as soon as seen by them. It is frequently the case that we are called in conclusion four or five days after the onset of the disease, and while at one time in my experience I operated on all of these cases as soon as seen, my mortality was astonishingly high. I am frank in saying that I now believe I have saved patients by not operating at this stage where before I lost them. Nothing has been said about the application of the ice bag and the administration of an occasional small dose of opium at this stage of the disease. Frequently the abscess will drain back through the bowel and for the time being relieve the situation. I admit that this is not the most desirable thing to occur, but it has unquestionably been the means of saving many lives. The sincerity of conviction compels me to repeat that my adherence to the practice as outlined is good surgery.

*Dr. Jones, (Closing)*.—I wish to thank those gentlemen who have discussed my paper this evening, for the many valuable points which they have brought out.

I wish I could feel as does Dr. McRae concerning the desperate cases, that it is better to delay than always operate on some of these cases. We have nothing to warrant that the abscesses are not just as likely to rupture into the peritoneal cavity as into some of the hollow viscera. I think the statistics of the larger hospitals show more recoveries now than formerly, largely because operation is done rather than wait and watch. Dr. Westmoreland brought out the fact that the great decrease in mortality in the desperate cases has come from early operation and less medical treatment. As Dr. Strickler said, if these bad cases die after the operation they would have died without it, and we would feel that all possible had not been done unless we operate.

In making up our statistics, I think it but fair to count these cases that die without the operation on the side of our mortality, provided we have the opportunity to do so and refuse.

I am hoping that some prognostic sign may be discovered which will always tell us how to proceed in the treatment of this very important condition, and until then I must remain on the side of those who advocate operation in every case unless some of the contra indications mentioned exist.

